# *Flavonifractor plautii* as a Next-Generation Probiotic Enhancing the NGP F/P Index in a Simulated Human Gut Microbiome Ecosystem

**DOI:** 10.3390/pharmaceutics17121603

**Published:** 2025-12-12

**Authors:** Md Sarower Hossen Shuvo, Sukyung Kim, Sujin Jo, Md Abdur Rahim, Indrajeet Barman, Mohammed Solayman Hossain, Youjin Yoon, Hanieh Tajdozian, Izaz Ahmed, Ali Atashi, GangWon Jeong, Ho-Seong Suh, JiMin You, Chaemin Sung, Mijung Kim, Hoonhee Seo, Ho-Yeon Song

**Affiliations:** 1 Department of Microbiology and Immunology, School of Medicine, Soonchunhyang University, Cheonan 31151, Chungnam, Republic of Korea; 2 K-Microbiome Institute, Soonchunhyang University, 22 Soonchunhyang-ro, Sinchang-myeon, Asan 31538, Chungnam, Republic of Korea; 3 Next-Generation Microbiome Training Center, Soonchunhyang University, 22 Soonchunhyang-ro, Sinchang, Asan 31538, Chungnam, Republic of Korea

**Keywords:** next-generation probiotics, *Flavonifractor plautii* PMC93, simulator of human intestinal microbial ecosystem, short-chain fatty acids, NGP F/P index, gastrointestinal microbiome

## Abstract

**Background/Objectives**: Traditionally consumed fermented foods and lactic acid bacteria (LAB)-based products have primarily been investigated for their nutritional and health-promoting benefits as dietary supplements. More recently, research has advanced toward exploring their therapeutic potential in pharmaceutical development. However, reliance on conventional LAB strains despite their established safety and efficacy has led to saturation at the strain level, underscoring the need for next-generation probiotics (NGPs) with novel therapeutic potential. In this context, we identified *Flavonifractor plautii* from human feces as a candidate NGP and investigated its effects on the human gut microbiota. **Methods**: Whole-genome sequencing revealed distinct genetic features that supported its uniqueness, and the strain was designated PMC93. A human gut microbial ecosystem simulator was used to administer *F. plautii* daily for one week, after which microbial community changes were evaluated using 16S rRNA gene-based metagenomic sequencing. **Results**: The administration did not induce significant changes in alpha or beta diversity, suggesting that *F. plautii* does not disrupt overall bacterial community structure, thereby supporting its microbial community safety. Taxonomic analysis demonstrated a significant increase in the *Firmicutes*-to-*Proteobacteria* ratio (NGP F/P index). The improvement surpassed that of conventional LAB treatments and was consistently maintained under supplementation with commonly encountered pharmaceutical compounds and nutrients. The shift was associated with an increase in short-chain fatty acid (SCFA)-producing beneficial taxa and a decrease in pro-inflammatory and potentially pathogenic groups. Functional outcomes, including elevated SCFA levels and downregulation of inflammation-related gene expression, further corroborated these compositional changes. The strain also demonstrated safety in in vivo models. **Conclusions**: Collectively, these findings suggest that strain PMC93 is a promising NGP candidate with substantial therapeutic potential for microbiota-associated health and disease modulation, particularly due to its ability to enhance the NGP F/P index.

## 1. Introduction

The global surge of interest in gut health has positioned probiotics and fermented foods as influential modulators of human physiology. The advantageous effects and safety profiles of lactic acid bacteria (LAB) and traditional probiotic strains have historically dominated the field of microbiome research and therapeutic use [1,2]. However, as research into the complex interactions between gut flora and overall health deepens, it has become clearer that the present probiotic repertoire may be approaching its functional threshold [3,4]. This saturation has led to an evolutionary shift toward the investigation of next-generation probiotics (NGPs), which have unique properties and therapeutic potential beyond those of conventional strains [5]. Unlike traditional probiotics, which often exert broad-spectrum effects, NGPs are being evaluated for their precision in modulating specific microbial populations and host pathways, offering a more targeted approach to microbiome-based interventions [6,7].

Among the most studied NGPs are *Akkermansia muciniphila*, *Faecalibacterium prausnitzii*, and *Bacteroides fragilis*, each demonstrating specialized roles in host physiology [8]. *A. muciniphila*, for example, has been associated with improved metabolic profiles and increased gut barrier integrity and is well-known for its ability to degrade mucin [9,10]. The well-known butyrate producer *F. prausnitzii* has potent anti-inflammatory properties and is frequently reduced in patients with inflammatory bowel disease [11]. Meanwhile, by producing polysaccharide A, which encourages the development of regulatory T cells, *B. fragilis* has demonstrated promise in regulating immune responses [12]. These NGPs exemplify the paradigm shift toward precision microbiome therapeutics, where strains are selected not merely for their survivability or general health benefits, but for their capacity to engage in specific biochemical and immunological interactions with the host [5,13]. This targeted functionality sets the stage for exploring lesser-known but equally promising candidates.

Building on this concept, *Flavonifractor plautii* has recently attracted interest due to its distinct metabolic features and potential roles in modulating immune responses [14]. *F. plautii* is an obligate anaerobe within the Clostridiales order, prevalent in the healthy human gut [15]. It is especially recognized for breaking down dietary flavonoids such as quercetin and catechin into bioactive metabolites that demonstrate anti-inflammatory and antioxidant properties [14,16]. Recent studies indicate that *F. plautii* correlates with a favorable gut microbial composition and could contribute to resolving inflammation by modulating T regulatory cell populations, enhancing IL-10 expression, and suppressing pro-inflammatory cytokines [17]. Furthermore, *F. plautii* appears to participate in bile acid transformation and the formation of short-chain fatty acids, highlighting its broader significance in host metabolic and immune regulation [18]. By contributing to these key metabolic pathways that shape the gut microbial balance, its activity directly relates to the *Firmicutes/Proteobacteria* (F/P) ratio—an established indicator of gut homeostasis, where higher F/P values denote a stable, SCFA-producing community and lower values indicate dysbiosis [19]. Within this context, *F. plautii* emerges as a compelling next-generation probiotic candidate capable of driving targeted improvements in gut health.

In order to elucidate the functional impact of *F. plautii* within the intestinal environment, a robust and adaptable model is necessary to assess the intricate interplay between gut bacteria and host physiology [20]. For these investigations, in vitro simulated gut ecosystems have emerged as informative platforms [20]. The simulator of the human intestinal microbial ecosystem represents a dynamic and validated system that closely replicates the physiological, biochemical, and microbiological features of the human gastrointestinal tract [21]. Its ability to maintain stable and reproducible microbial communities under controlled conditions makes it particularly suited for testing the effects of dietary compounds, pharmaceuticals, or probiotic strains on microbiota composition and function [22].

Utilizing a standard simulation model, this study aims to characterize the modifications in gut microbial community structure following *F. plautii* supplementation. Through rigorous in vitro and in vivo validation, we investigated the resulting shifts in microbial metabolic pathways to better understand how *F. plautii* influences intestinal metabolic activity. The findings reinforce the functional importance of *F. plautii* and highlight its potential as a next-generation probiotic for microbiome-based interventions.

## 2. Materials and Methods

### 2.1. Clinical Stool-Derived Strain Isolation and Cell Bank Development

The experiment was conducted to isolate an obligate commensal bacterium with potential as an NGP, expected to surpass existing NGP strains in probiotic efficacy. Accordingly, stool samples were collected from 17 healthy volunteers at the Hospital of Soonchunhyang University in Seoul, Republic of Korea. Details of the bacterial isolation process have been reported earlier [23]. The study was conducted in accordance with the principles outlined in the Helsinki Declaration. Approval was obtained from the Institutional Review Board (IRB) for Human Research of Soonchunhyang University Seoul Hospital (IRB number: SCH 2019-12-004). Stool samples were promptly processed under stringent anaerobic conditions to enable the isolation of gut commensal bacteria using an anaerobic chamber (The Baker Co., Sanford, ME, USA), diluted, and enriched in Brain Heart Infusion (BHI) media (Kisan Bio, Seoul, Republic of Korea). Primary isolation was performed on BHI agar (Kisan Bio, Seoul, Republic of Korea), followed by sub-culturing of individual colonies. Bacterial growth was quantified at OD600 using a spectrophotometer (DR 1900, Hach, Loveland, CO, USA) prior to identification by 16S rRNA sequencing (Biofact, Daejeon, Republic of Korea). A Master Cell Bank (MCB) was established after sequence verification to ensure uniformity and reproducibility, with isolates stored in cryogenic storage.

### 2.2. Identification of F. plautii by 16S rRNA Gene Sequencing

The 16S rRNA gene sequencing technique was initially used to identify the *F. plautii* bacteria. The identification procedure was performed according to the previously published report [24]. The pair of primers 27F (5′-AGA GTT TGA TCC TGG CTC AG-3′) and 1492R (5′-GGT TAC CTT GTT ACG ACT T-3′) was used for PCR amplification after DNA extraction [25]. The amplified PCR product was then purified and sequenced using an ABI PRISM 3730XL DNA analyzer (Applied Biosystems, Foster City, CA, USA). The sequencing data were then compared to the National Center for Biotechnology Information (NCBI) GenBank database using BLAST (version 2.13.0) (basic local alignment search tool).

### 2.3. Whole-Genome Sequencing of the F. plautii PMC93

Whole-genome sequencing (WGS) of the PMC93 was performed according to a previously published study [26]. A QIAamp DNA Mini Kit (Qiagen, Hilden, Germany) was used to extract gDNA following PBS washes of the bacterial culture. Chunlab constructed PacBio libraries and carried out whole-genome sequencing. Using a g-tube (Covaris, Woburn, MA, USA), genomic DNA was divided into 10 kb and purified. Using the SMRTbell Template Prep Kit 1.0 (PacBio, Menlo Park, CA, USA), SMRTbell adapters were ligated to the blunt end after the ends were fixed. After that, the library was sequenced in an 8-well SMART Cell v3 of PacBio RSII (PacBio) using PacBio P6C4 chemistry. PacBio SMRT Analysis 2.3.0 was used to assemble PacBio sequencing data using the HGAP2 procedure. After that, a Circlator 1.4.0 (Sanger Institute, Hinxton, UK) was used to circularize the genome. Prodigal 2.6.2 [27] was used to predict protein coding sequences (CDSs), which were then categorized based on their roles in relation to orthologous groups (EggNOG; http://eggnogdb.embl.de) (accessed on 1 July 2025). tRNAscan-SE 1.3.1 was used to look for genes that encode tRNAs [28]. The Rfam 12.0 database was used to conduct covariance model searches for rRNAs and other noncoding RNAs [29]. The OrthoANIu algorithm-based Average Nucleotide Identity (ANI) calculator was utilized to compare the genome sequences of prokaryotic organisms [30].

### 2.4. Preparation of the F. plautii PMC93

PMC93 was cultured in 30 mL of BHI broth and incubated at 37 °C in an anaerobic chamber (The Baker Co., Sanford, ME, USA) for 24 h. Cultures were adjusted to an OD of 1.0 at 600 nm using a spectrophotometer. After that, centrifugation was performed at 4000 rpm for 30 min using a centrifuge machine (Hanil Scientific, Gimpo, Republic of Korea), followed by washing with 0.85% NaCl solution to remove medium components. Then, the pellet was suspended using 1 mL of 0.85% NaCl solution. The prepared PMC93 suspension was subsequently utilized for experimental testing.

### 2.5. Simulation of the Human Gut Microbiome for Evaluating the Effects of F. plautii PMC93

This study employed the Human Gut Microbiome Simulation (HGMS; adapted from the SHIME^®^, ProDigest, Ghent, Belgium), a dynamic in vitro model that replicates the entire human gastrointestinal tract [31,32]. In the HGMS, each unit consists of five serially connected vessels, simulating distinct segments of the human gut: the stomach (ST), small intestine (SI), ascending colon (AC) (working volume = 500 mL), transverse colon (TC) (working volume = 800 mL), and descending colon (DC) (working volume = 600 mL). The system was inoculated with fresh fecal microbiota obtained from a healthy adult donor with no antibiotic use in the preceding three months. The freshly collected stool was processed under anaerobic conditions, prepared as a 5% (*w/v*) fecal suspension, and introduced into the colon vessels. The ST and SI compartments operate on a fill-and-draw principle to mimic the dynamic processes of food uptake and digestion. Specifically, 140 mL of simulator nutritional medium was introduced into the ST vessels three times daily (at 5:00 PM, 1:00 AM, and 9:00 AM). Concurrently, 60 mL of pancreatic/bile juice was introduced into the SI vessels three times daily (at 6:30 PM, 2:30 AM, and 10:30 AM). Following defined retention periods, the contents of these reactors are emptied, replicating the physiological transit observed in the upper gastrointestinal tract. The temperature of all vessels was consistently maintained at 37 °C using a water bath. The pH within each colon vessel (AC, TC, and DC) was continuously monitored and automatically adjusted to physiological levels using 0.5 M NaOH or HCl. Throughout the experimental period, each vessel was continuously stirred at 300 rpm to ensure homogeneity and proper mixing of the microbial communities and luminal contents. In the comparative setup, one unit served as the untreated control, while the other administered a daily dose of 10 mL PMC93 at a concentration of ${1\;\times 10}^{9}$ CFU/mL.

### 2.6. Ex Vivo Multi-Treatment Assays for Gut Microbiome Modulation by Conventional Probiotics and Common Therapeutic Agents

In addition to the standard gut ecosystem simulator-based intervention, an ex vivo multi-treatment Human Gut Microbiome Simulation (ex vivo MT-HGMS) was incorporated to evaluate microbial responses to probiotic supplementation further. Samples were collected aseptically from the gut microbiome simulator and immediately subjected to ex vivo MT-HGMS. These samples were incubated at 37 °C for five days under continuous agitation on a shaker (Biofree, Seoul, Republic of Korea) to simulate intestinal motility and maintain homogenous microbial exposure to the test treatments (at a concentration of ${1\;\times 10}^{9}$ CFU/mL). The experimental treatments included the novel probiotic strain PMC93 as well as some conventional lactic acid bacterial strains: *Lactiplantibacillus pentosus*, *Latilactobacillus sakei*, *Latilactobacillus curvatus*, and *Leuconostoc mesenteroides*. They were isolated from kimchi, a traditional Korean fermented food. Samples were homogenized and serially diluted, followed by plating on de Man, Rogosa, and Sharpe (MRS) agar (BD Difco, Franklin Lakes, NJ, USA). After incubation, morphologically distinct colonies were selected and purified. Genomic DNA was extracted from the isolates, and species identification was performed by 16S rRNA gene sequencing (Table S1). To investigate potential synergistic or modulatory interactions, PMC93 was further evaluated in combination with four conventional therapeutic agents chosen for their relevance to gut health and systemic physiology: Vitamin D (CNS Pharm, Seoul, Republic of Korea), Albendazole (Boryung Pharma, Seoul, Republic of Korea), Famotidine (Daewoong Pharma, Seoul, Republic of Korea), and Finasteride (Dongwha Pharma, Seoul, Republic of Korea). Each drug was evaluated (at a concentration of 1 mg/mL) in combination with either the selected probiotics or PMC93 to enable comparative analysis of their effects on gut microbial composition. This ex vivo MT-HGMS enabled the evaluation of how PMC93 affects gut microbial composition in comparison to established probiotic and therapeutic interventions.

### 2.7. 16S rRNA Gene-Based Metagenomics Analysis

Metagenomics analysis was performed in accordance with the procedures described previously by our team [33]. Metagenomic DNA was extracted from the collected samples using a QIAamp DNA Mini Kit (Qiagen, Hilden, Germany) and quantified with a Qubit-4 fluorometer (Thermo Fisher Scientific, Waltham, MA, UK). DNA integrity and quality were confirmed via 0.8% agarose gel electrophoresis, and samples were stored at −20 °C until further processing. The V4 hypervariable region of the 16S rRNA gene was amplified using specific primers (Forward: TCG TCG GCA GCG TCA GAT GTG TAT AAG AGA CAG CCT ACG GGN GGC WGC AG; Reverse: GTC TCG TGG GCT CGG AGA TGT GTA TAA GAG ACA GGA CTA CHV GGG TAT CTA ATCC) and PCR with KAPA HiFi HotStart ReadyMix (Kapa Biosystems, Wilmington, MA, USA), followed by cleanup with AMPure XP beads (Beckman Coulter, Brea, CA, UK) [34,35]. Libraries were prepared using the Nextera XT DNA Library Prep Kit (Illumina, San Diego, CA, USA) and sequenced on the Illumina iSeq100 platform (Illumina, San Diego, CA, USA). Sequence analysis was performed using the Quantitative Insights Into Microbial Ecology (QIIME) software (version 1.9.1) [36]. A 97% similarity criterion was used to assign sequences to operational taxonomic units (OTUs). The RDP classifier was used to assign taxonomic data to representative sequences for every OTU [37]. The Human Microbiome Database was then used to map these sample sequences to the taxonomy hierarchy, from phylum to family. For the taxonomy assignment, a Bayesian method with a 97% confidence level was used. Relative abundance data that met normality assumptions were analyzed using unpaired *t*-tests, whereas Wilcoxon tests were used to analyze sample datasets whose normality could not be assumed. α-diversity indices, such as observed Chao1, Fisher, Simpson, and Shannon indices, were computed to evaluate the richness and diversity of bacterial communities in the samples [37,38,39,40]. Using indices like Bray–Curtis and Jansen–Shannon, the variation in bacterial communities between samples (β-diversity) was examined [33,41,42]. Permutational multivariate analysis of variance (PERMANOVA) was employed to assess the statistical significance of beta diversity.

### 2.8. Short-Chain Fatty Acid Quantification

Short-chain fatty acids (SCFAs) were measured following the procedure described in a previous study [43]. SCFAs were measured from samples of a simulated gut ecosystem collected on Days 0, 4, and 7 during continuous PMC93 feeding. Each sample involved mixing 1 mL of HGMS effluent with 2.5 g sodium chloride (NaCl) and 1 mL of 2% sulfuric acid in a sealed headspace vial. The mixtures were analyzed using a TurboMatrix Headspace Sampler (PerkinElmer, Waltham, MA, USA). SCFAs were identified using gas chromatography–mass spectrometry (GC-MS) on a PerkinElmer Clarus 690 GC system paired with a Clarus SQ8 MS detector (PerkinElmer). Compounds were separated with an Elite-FFAP capillary column (30 m × 0.25 mm i.d., 0.25 µm film thickness). The injector temperature was set at 250 °C, with an injection volume of 0.16 mL in split mode (5:1). Helium served as the carrier gas at a flow rate of 16 mL/min. The oven program increased the temperature from 120 °C to 200 °C at 5 °C/min. The ion source operated at 250 °C, and data collection was performed in full scan mode. Calibration curves were prepared using standard solutions of acetic, propionic, butyric, and valeric acids (1–100 mg/L). SCFA identification in samples relied on comparison of retention times and mass spectra to those of the standard compounds.

### 2.9. Gene Expression Analysis of Intestinal Tissues

Gene expression was assessed using methods previously established by our group [44]. To determine the effect of PMC93 on inflammatory responses, gene expression levels were assessed by quantitative real-time PCR (qRT-PCR). Nine-week-old female Balb/C mice received oral administration of PMC93 (concentration: ${5\;\times 10}^{8}$ CFU/mL; administration volume: 200 µL per mouse) over two weeks. Following the treatment, gut tissues were harvested and subjected to mRNA extraction using the RNeasy Mini Kit (Qiagen, Hilden, Germany), strictly following the manufacturer’s protocol. The mRNA concentration was measured using a Qubit Fluorometer (Invitrogen, Carlsbad, CA, USA) in conjunction with the Qubit RNA Assay Kit (Thermo Fisher Scientific, Waltham, MA, USA). Complementary DNA (cDNA) synthesis from the purified mRNA was conducted utilizing a cDNA synthesis kit (Bio-Rad, Hercules, CA, USA). Quantitative real-time PCR was then performed with the SYBR Green Supermix Kit (Bio-Rad, USA) on a CFX96 Real-Time PCR Detection System (Bio-Rad, USA), in accordance with the manufacturer’s instructions. The analysis focused on the expression levels of key pro-inflammatory cytokines (COX-2, iNOS, TNF-α, IL-6, IL-1β, and IL-12). The beta-actin gene served as the internal control (housekeeping gene). Relative mRNA expression was calculated using the comparative Ct ($2^{-\Delta \Delta Ct}$) method as described previously [45]. The primer sequences used in the amplification reactions are provided in Supplementary Table S3.

### 2.10. Acute Oral Toxicity Study of F. plautii PMC93 in Mice

The acute oral toxicity assay was conducted as described previously [46,47]. A repeated oral toxicity study was conducted to assess the safety profile of PMC93 in mice. Nine-week-old female Balb/C mice were randomly assigned to two groups: one group was administered PMC93 (concentration: ${5\;\times 10}^{8}$ CFU/mL; administration volume: 200 µL per mouse) orally for two weeks, while the control group received distilled water. Animals were housed under controlled environmental conditions and provided food and water ad libitum. Throughout the study, mice were monitored daily for clinical signs of toxicity, mortality, and alterations in body weight. All experimental protocols complied with institutional and national regulations for laboratory animal care, following IACUC approval (approval number: SCH25-0028).

## 3. Results

### 3.1. Isolation and Characterization of F. plautii PMC93 and Cell Bank Development

**Figure 1:**
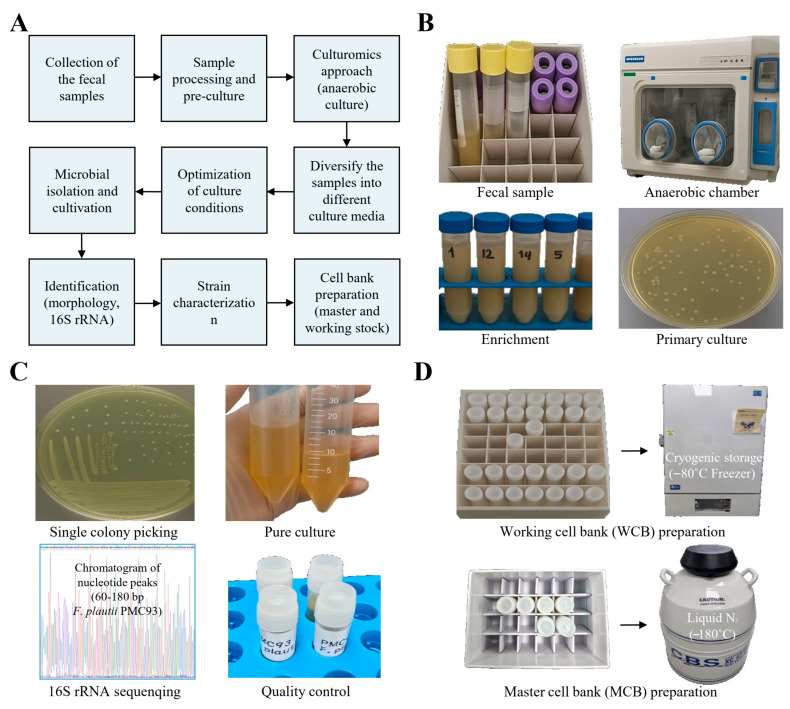
Workflow for culturomics-based isolation, identification, and cell banking of *F. plautii*. Flowchart of the culturomics-based novel strain development strategy (**A**), and the results are presented sequentially. The process began with anaerobic culture of fecal samples (**B**). The collection of human fecal samples was followed by sample processing, enrichment, and anaerobic culture in an anaerobic chamber to obtain primary cultures. Next, microbial isolation and identification experiments were performed (**C**). Microbial isolation and cultivation were performed through single colony picking, leading to pure culture development. The isolate was then subjected to identification by 16S rRNA sequencing and strain characterization. After confirming the strain as *F. plautii*, the culture was optimized under different conditions and subsequently used for cell bank preparation (**D**). Finally, both a working cell bank (WCB) was preserved at −80 °C, and a master cell bank (MCB) was preserved in liquid nitrogen at −180 °C for long-term storage.

A novel strain of *F. plautii* was isolated from human fecal samples using a culturomics-based approach, identified by 16S rRNA sequencing, and preserved through the establishment of master and working cell banks (Figure 1 and Table 1). A flowchart summarizing the culturomics-based novel strain development strategy is presented in Figure 1A. The workflow commenced with the collection, enrichment, and anaerobic culturing of fecal samples in an anaerobic chamber, which facilitated the development of primary cultures (Figure 1B). Microbial colonies were then isolated, and single colonies were picked to establish pure cultures (Figure 1C). The isolate was subjected to molecular identification by 16S rRNA sequencing, confirming the strain as *F. plautii*. Further optimization of culture conditions allowed successful strain characterization and large-scale cultivation. For long-term preservation, a working cell bank (WCB) was prepared at −80 °C, while a master cell bank (MCB) was established using liquid nitrogen storage at −180 °C (Figure 1D). This systematic workflow ensured both the accurate identification and stable preservation of *F. plautii* for future application as a next-generation probiotic candidate.

### 3.2. Whole-Genome Sequencing and Functional Insights into F. plautii PMC93

**Figure 2:**
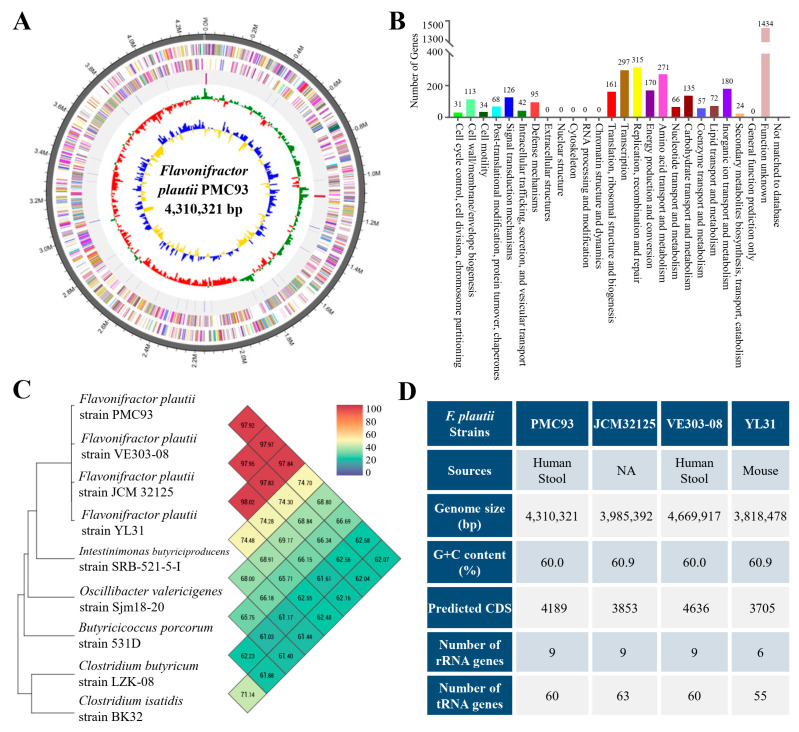
Whole-genome sequencing of *F. plautii* PMC93. The genomic structure and comparative features of strain PMC93 were analyzed to elucidate its taxonomic position, functional potential, and relationship with closely related strains. Single circular map of PMC93 (**A**) presents the composition of the whole genome (4,310,321 bp). Color-coded Cluster of Orthologous Groups (COG) functional categories of forward and reverse strands are presented from the outer edge of the circle. tRNA and rRNA genes are indicated in red and blue, respectively. The inner rings display GC content (green/red) and GC skew (yellow/blue), highlighting genomic composition and strand bias. Functional classification of predicted genes according to COG categories (**B**) revealed the distribution of metabolic, cellular, and information-processing functions. Orthologous Average Nucleotide Identity (OrthoANI) analysis (**C**) compared PMC93 with closely related species. The PMC93 genome shares more than 97% ANI with *F. plautii* strain JCM32125, VE303-08, and YL31, exceeding the threshold (96%) for species delineation. Comparative genomic features of PMC93, JCM32125, VE303-08, and YL31 (**D**) show differences in isolation source, genome size, G+C content, predicted coding sequences (CDSs), and the number of rRNA and tRNA genes.

The study conducted a WGS analysis for the candidate strain (Figure 2). The genome comprises a single circular chromosome of 4,310,321 bp, containing 4189 coding DNA sequences (CDSs) (Figure 2A). Functional annotation classified 3691 predicted proteins into Cluster of Orthologous Groups (COG) categories (Figure 2B). Of these, 2257 proteins were assigned specific biological functions, while 1434 CDSs matched conserved proteins of unknown function in other organisms. Whole-genome similarity assessment was performed using the OrthoANI method for strains with high 16S rRNA sequence similarity (Figure 2C). The strain showed 97.92%, 97.95% and 98.02% nucleotide identity to *F. plautii* strain VE303-08, JCM32125, and YL31, respectively. Its similarity to other genera, such as *Intestinimonas butyriciproducens*, *Oscillibacter valericigenes*, and *Butyricicoccus porcorum*, was 74.48%, 68.0%, and 65.75%, respectively, which were all below 80%. These results strongly suggest that the newly discovered strain PMC93 is *F. plautii*. A comparative genomic analysis between *F. plautii* strain PMC93, JCM32125, VE303-08, and YL31 highlighted variations in genome length, G+C content, CDS number, and rRNA/tRNA gene counts (Figure 2D). For comparative genomic analysis in this study, publicly available datasets from the NCBI BioProject repository were utilized. Specifically, the genomes of *F. plautii* strain JCM32125 (BioProject: PRJNA603247), VE303-08 (BioProject: PRJNA755324), and YL31 (BioProject: PRJNA224116) were included. The accession number of PMC93 in the NCBI database is PRJNA1372262.

### 3.3. Operation of Human Gut Microbiome Simulation Systems to Evaluate the NGP Potential of F. plautii PMC93

**Figure 3:**
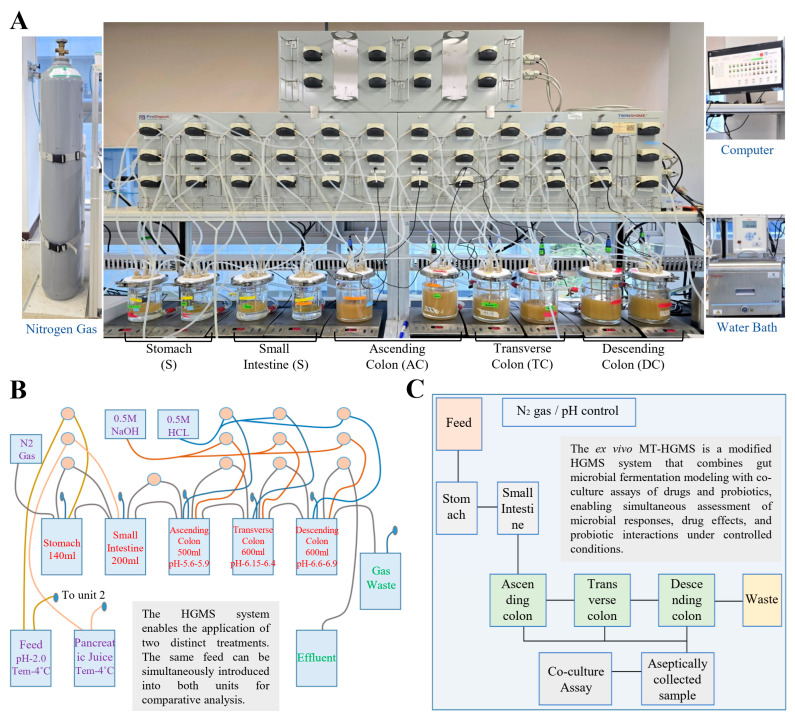
Human gut microbiome simulation systems. The figure depicts the HGMS-based experimental setup and its modifications for evaluating microbial interactions: (**A**) The experimental evaluation of PMC93 was conducted using the twin unit HGMS. This advanced in vitro model simulates the human gastrointestinal tract, including the stomach (S), small intestine (SI), and three colon compartments: the ascending colon (AC), transverse colon (TC), and descending colon (DC). As the HGMS consists of two identical units, one unit was maintained as the untreated control, while the other was used for PMC93 treatment. A schematic of the twin unit HGMS (**B**) highlights the flow of materials and treatments across different compartments. The color-coded lines represent the transfer pathways. Schematic diagram of the ex vivo MT-HGMS (**C**) highlights the remodeling of the simulator. It combines the standard HGMS with an in vitro co-culture assay. In this modified system, samples can be exposed to multiple treatments, including control, PMC93, probiotics, and drug combinations, simultaneously to assess their impact on the gut microbiome.

To investigate the modulatory effects of PMC93 on the human gut microbiota, a Human Gut Microbiome Simulation (HGMS) platform was utilized that simulates the physiological conditions of the gastrointestinal tract. Figure 3 presents the experimental setup and its enhancements for microbial interaction studies. The twin unit HGMS system (Figure 3A) was employed to replicate the stomach (S), small intestine (SI), and three colon compartments—ascending colon (AC), transverse colon (TC), and descending colon (DC)—under controlled pH and temperature conditions. Each compartment was maintained at physiologically relevant pH ranges (AC: 5.6–5.9, TC: 6.1–6.4, and DC: 6.6–6.9) and continuously fed with nutrient medium and pancreatic juice at 4 °C. Nitrogen gas was used to maintain anaerobic conditions, and effluent samples were collected daily for microbial analysis. The schematic of the HGMS system (Figure 3B) illustrates the flow of feed, digestive fluids, and microbial waste across compartments. This setup enabled dynamic monitoring of microbial composition and metabolite production over time. To enhance resolution, the ex vivo multi-treatment Human Gut Microbiome Simulation (ex vivo MT-HGMS) system (Figure 3C) was developed by coupling the standard HGMS with an in vitro co-culture assay. This allowed for simultaneous exposure of microbial communities to multiple treatment conditions: control (no intervention), PMC93, conventional probiotics, and PMC93–drug combinations.

### 3.4. Insights into Microbial Community Structure and Diversity Using HGMS

**Figure 4:**
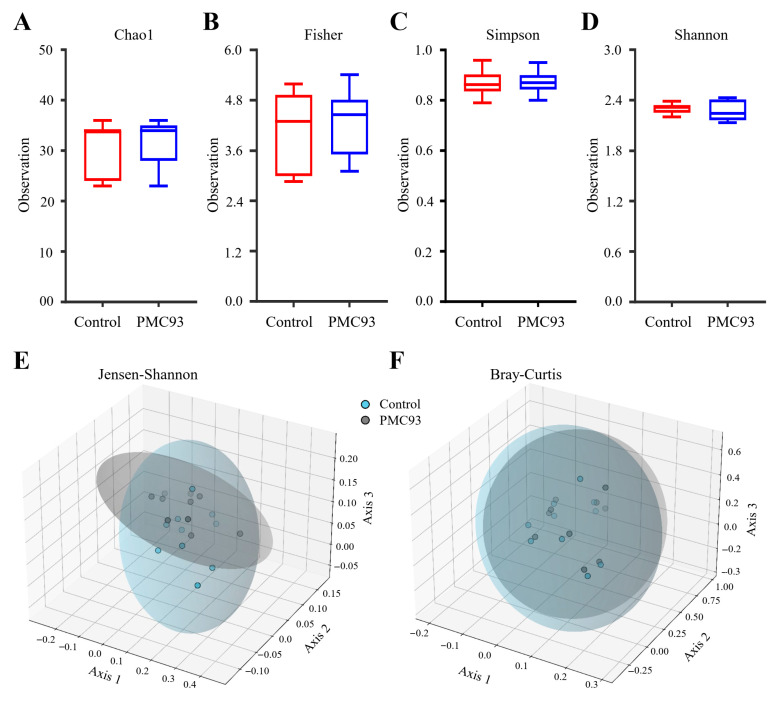
Alpha and beta diversity profiling of gut microbiota in HGMS. Alpha diversity metrics were evaluated to determine microbial richness and diversity. Species richness was quantified using the (**A**) Chao1 and (**B**) Fisher indices, while diversity was assessed by the (**C**) Simpson and (**D**) Shannon indices. Data are displayed as boxplots, with box edges denoting the first and third quartiles and the median indicated by a horizontal line. Beta diversity was evaluated by principal coordinate analysis (PCoA) using the (**E**) Jensen–Shannon divergence and (**F**) Bray–Curtis Index. Colored ellipses represent 95% confidence intervals for each group. No significant differences were found between the control and PMC93 groups.

In HGMS, alpha diversity analysis identified some alterations between the control and PMC93 groups (Figure 4). The PMC93 group showed a moderate elevation in species richness, as indicated by higher Chao1 and Fisher index values relative to the control group (Figure 4A,B). In contrast, the Shannon and Simpson diversity indices, which quantify both species richness and evenness, demonstrated similar results for the two groups (Figure 4C,D). Although PMC93 modestly increased species richness, the overall diversity of the microbial community was not significantly impacted. Beta diversity was assessed using Jensen–Shannon (Figure 4E) and Bray-Curtis metrics (Figure 4F). In the 3D principal coordinates analysis (PCoA) plots, the control and PMC93 groups formed separate clusters, showing overlaps. This group separation was statistically validated by PERMANOVA analysis, with both the Jensen–Shannon and Bray–Curtis indices demonstrating no statistically significant group differences (*p* = 0.60 and 0.931, respectively).

### 3.5. Taxonomic Profiling and Comparative Analysis of Microbial Communities in HGMS

**Figure 5:**
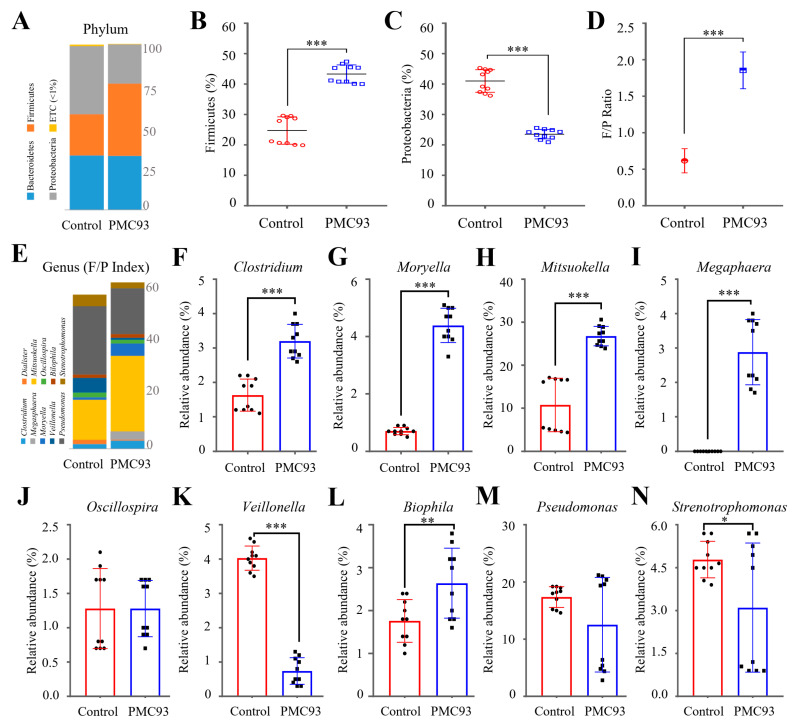
Comparative taxonomic composition of gut microbiota in HGMS. The microbial community structure was assessed to evaluate the impact of PMC93 supplementation: (**A**) Phylum-level distribution showing major phyla: *Firmicutes*, *Bacteroidetes*, and *Proteobacteria*. (**B**) Significant expansion of *Firmicutes*, and (**C**) reduction in *Proteobacteria* was observed following PMC93 treatment. (**D**) F/P ratio, demonstrating a substantial shift after PMC93 treatment. (**E**) Genus-level landscape, illustrating taxa contributing to the elevated F/P Index. A significant increase in the abundance of (**F**) *Clostridium*, (**G**) *Moryella*, (**H**) *Mitsuokella*, and (**I**) *Megasphaera* was observed, whereas (**J**) *Oscillospira* remained stable, and (**K**) *Veillonella* showed a decline following the administration of PMC93 in *Firmicutes*. In contrast, (**L**) an increase in *Bilophila*, as well as (**M**) a decrease in the abundance of *Pseudomonas* and (**N**) *Stenotrophomonas*, were observed following PMC93 treatment for *Proteobacteria*. For each experimental group, ten biological samples were analyzed. The Wilcoxon rank-sum test was used to evaluate group differences. No taxa exceeding 1% relative abundance exhibited statistically significant variations across all ranks. (* *p* < 0.05; ** *p* < 0.01; *** *p* < 0.001).

Taxonomic composition was evaluated at several hierarchical ranks- Phylum, Class, Order, Family, and Genus, to investigate variations in microbial abundance and diversity in HGMS. The data reveal statistically significant differences across the analyzed groups. Raw data are presented in Table S2, while taxa with relative abundances exceeding 1% and displaying significant group differences were extracted in Table 2 and Figure 5. At the phylum level, distinct differences were detected between the control and PMC93 groups (Figure 5A). *Firmicutes* exhibited a markedly higher abundance in the PMC93 group (43.3%) compared to the control group (24.7%) (*p* = 0.0004) (Figure 5B). In comparison, *Proteobacteria* were more prevalent in the control group (41.0%) than in the PMC93 group (23.5%) (*p* = 0.0004) (Figure 5C). The proportion of *Bacteroidetes* did not differ significantly between the two groups (*p* = 0.5390). The study reveals a significantly higher *Firmicutes*-to-*Proteobacteria* (F/P) ratio (*p* < 0.001), which indicates a shift in the microbial community toward *Firmicutes* dominance (Figure 5D). At the class level, PMC93 supplementation resulted in a pronounced enrichment of *Firmicutes*-associated taxa (Table 2), particularly *Clostridia*, which increased from 23.7% in the control group to 43.1% in the PMC93 group (*p* = 0.0004). Within this class, the order *Clostridiales* showed a parallel increase (23.7% to 43.1%, *p* = 0.0004), with notable enrichment of the families *Veillonellaceae* (31.6% vs. 16.2%) and *Lachnospiraceae* (9.3% vs. 4.2%) (both *p* < 0.001). Conversely, several *Proteobacteria*-associated taxa were markedly reduced following PMC93 treatment (Table 2). *Gammaproteobacteria* abundance decreased from 39.4% in the control group to 20.0% in the PMC93 group (*p* = 0.0004), primarily driven by a sharp decline in the order *Enterobacteriales* (16.6% to 1.5%, *p* = 0.0004) and its dominant family *Enterobacteriaceae* (16.6%, *p* < 0.001). The orders *Pseudomonadales* and *Xanthomonadales* showed no significant changes, whereas *Alphaproteobacteria* (0.5% to 2.1%, *p* = 0.0004) and *Deltaproteobacteria* (0.9% to 1.3%, *p* = 0.0376) exhibited modest increases. Figure 5E illustrates the genus-level microbial composition that contributed to the F/P index for the control and PMC93 groups. The PMC93 group displayed a greater representation of *Firmicutes*-associated genera (Figure 5F–I), such as *Clostridium* (3.2%), *Moryella* (4.4%), *Mitsuokella* (26.8%), and *Megasphaera* (2.9%), which were significantly more abundant compared to the control group (all *p* < 0.001). *Oscillospira* showed no significant change between groups (Figure 5J), and *Veillonella* was significantly reduced in the PMC93 group (*p* < 0.001, Figure 5K). In contrast, the control group harbored significantly higher levels of several genera associated with *Proteobacteria* dominance, including *Pseudomonas* and *Stenotrophomonas*, which were decreased in the PMC93 group (*p* < 0.05) (Figure 5M,N). A modest but significant elevation in *Bilophila* (*p* < 0.01) (Figure 5L) was observed. These compositional differences visually reflect the enrichment of beneficial *Firmicutes* and the reduction in potentially pathogenic *Proteobacteria*, in line with the significantly higher F/P ratio observed in the PMC93 group.

### 3.6. Comparative Microbiota-Modulating NGP Effects of F. plautii PMC93 in Ex Vivo MT-HGMS

**Figure 6:**
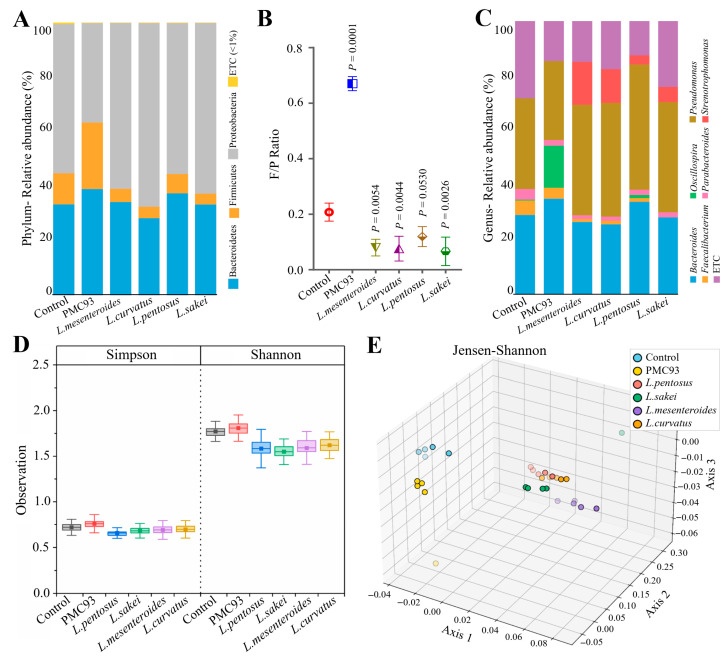
Ex vivo MT-HGMS analysis of microbiome modulation. PMC93 induced distinct microbial profiles compared to other conventional probiotics. Taxonomic composition of the gut microbiota at the phylum level (**A**) showing the distribution of *Firmicutes*, *Bacteroidetes*, and *Proteobacteria* in each treatment group: control, PMC93, and conventional probiotics (*L. pentosus*, *L. sakei*, *L. curvatus*, and *L. mesenteroides*). F/P ratio across groups, with the PMC93 group (**B**) showing a significantly higher ratio compared to the control and all other treatments. The *p*-values, determined using unpaired *t*-tests, are indicated above each comparison. Genus-level composition (**C**) displays the relative abundance of dominant genera. PMC93 treatment is associated with reduced abundance of opportunistic genera from *Proteobacteria*, such as *Pseudomonas* and *Stenotrophomonas*, and enrichment of beneficial taxa from *Firmicutes* (*Oscillospira* and *Faecalibacterium*). Alpha diversity (**D**) was measured using Simpson and Shannon indices as indicators of species diversity. The results are shown as boxplots, depicting the median and the first and third quartiles. There were no statistically significant changes in alpha diversity among groups. Beta diversity (**E**) was measured using PCoA based on Jensen–Shannon divergence.

Comparative microbiota modulating the NGP effects of PMC93 were analyzed in ex vivo MT-HGMS. Compared to both the control and the conventional probiotic groups (*L. mesenteroides*, *L. curvatus*, *L. pentosus*, and *L. sakei*), the PMC93 treatment group exhibited distinct alterations in microbial community composition (Figure 6). At the phylum level, PMC93 markedly increased the proportion of *Firmicutes* while reducing *Proteobacteria* relative to all other groups (Figure 6A), resulting in the highest F/P ratio (0.72, *p* < 0.0001) among all treatments (Figure 6B). In contrast, the conventional probiotic groups exhibited low F/P ratios ranging from 0.12 to 0.22. At the genus level, *Bacteroides* was most prevalent in the PMC93 group (35.0%), whereas it accounted for 28.9% in the control and ranged from 25.5% to 34.0% in all conventional probiotic groups (Figure 6C). *Oscillospira* was markedly enriched in the PMC93 group (15.4%) but remained nearly undetected in all other groups (0.0–1.3%). *Pseudomonas* abundance in the PMC93 group (28.8%) was reduced relative to both the control (33.0%) and the other probiotic groups, which had levels between 40.9% and 45.7%. *Faecalibacterium* showed a modest decrease in PMC93 (3.9%) compared to the control (5.2%); however, its abundance exceeded that found in the probiotic groups (0.0–1.3%). *Stenotrophomonas* was undetectable in both the control and PMC93 groups, but it was identified in all probiotic groups, reaching peak abundance in *L. mesenteroides* (15.9%). The distinct enrichment of beneficial *Firmicutes* genera in PMC93 and the corresponding reduction in potential pathobionts highlight a favorable restructuring of the gut microbiota compared to both the control and conventional probiotics. Alpha diversity analysis employing the Simpson and Shannon indices (Figure 6D) indicated that all treatment groups, including PMC93 and probiotic strains (*L. pentosus*, *L. sakei*, *L. mesenteroides*, and *L. curvatus*), maintained diversity metrics closely resembling those of the control. The Simpson index demonstrated that all probiotic strain treatment groups exhibited slightly lower diversity compared to the control, whereas the PMC93 group displayed a marginal increase. The Shannon index results were comparable to those of the Simpson index. All groups receiving *Lactobacillus* strains exhibited somewhat decreased diversity relative to the control, but the PMC93 group demonstrated a modestly higher value. Beta diversity assessed through 3D principal coordinates analysis (PCoA) (Figure 6E) revealed discrete clustering among the groups. Separate clusters were observed for the control and PMC93 groups, while varying degrees of separation were found among samples from each probiotic strain.

### 3.7. Gut Microbiota Remodeling by Combined Therapeutic Treatments in Ex Vivo MT-HGMS

**Figure 7:**
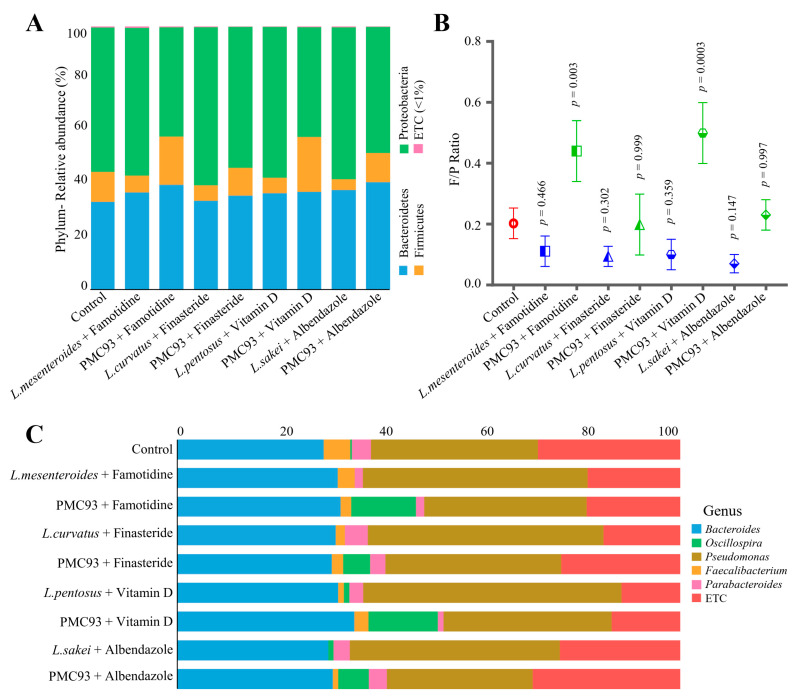
Effects of combined therapeutics on gut microbial composition in ex vivo MT-HGMS. The influence of combinational drug–probiotic treatments on gut microbiota was summarized at different taxonomic levels. Stacked bar (**A**) showing the relative abundance of dominant bacterial phyla *Firmicutes*, *Bacteroidetes*, *Proteobacteria*, and others (<1%) across experimental groups, including various drug treatments (Famotidine, Finasteride, Vitamin D, and Albendazole) administered with the probiotic strains (*L. pentosus*, *L. sakei*, *L. curvatus*, and *L. mesenteroides*), and PMC93. For each experimental group, three biological samples (N = 3) were analyzed. Changes in the F/P ratio (**B**) across groups (mean ± standard deviation) were shown with identified *p*-values by unpaired *t*-tests. Significant increases were observed in selected PMC93-treated groups. The relative abundance of dominant gut microbial genera (**C**) was presented across the nine experimental conditions. PMC93 co-treatment consistently enhanced the relative abundance of beneficial genera of *Firmicutes*, such as *Faecalibacterium* and *Oscillospira*, while the abundance of *Pseudomonas* (*Proteobacteria*) was reduced in the presence of PMC93.

The impact of PMC93 was further assessed in combination with conventional therapeutic agents such as Vitamin D, Albendazole, Famotidine, and Finasteride—each administered alongside either selected conventional probiotics (*L. pentosus*, *L. sakei*, *L. curvatus*, and *L. mesenteroides*) or PMC93 (Figure 7) in ex vivo MT-HGMS. At the phylum level (Figure 7A), PMC93-containing treatments consistently increased *Firmicutes* and reduced *Proteobacteria* relative to probiotic–drug combinations, leading to markedly higher F/P ratios (Figure 7B). Notably, PMC93 + Finasteride (*p* = 0.003) and PMC93 + Vitamin D (*p* = 0.0003) achieved significantly higher F/P ratios compared to their respective probiotic–drug groups. At the genus level (Figure 7C), PMC93 combinations led to increased relative abundance of *Bacteroides*, a key commensal genus associated with intestinal health, with the highest levels observed in the PMC93 + Vitamin D (34.7%) and PMC93 + Albendazole (35.3%) groups. Furthermore, *Oscillospira*, a known producer of beneficial SCFAs, was enriched in PMC93-treated groups, reaching 13.4% in the PMC93 + Famotidine group, compared to near absence in the control and other probiotic–drug conditions. In contrast, probiotic–drug treatments often led to an expansion of potentially opportunistic genera from the *Proteobacteria*, such as *Pseudomonas*, whereas PMC93 treatments reduced this. This suggests that PMC93 helps suppress potentially opportunistic bacteria. These findings highlight the superior modulatory capacity of PMC93 in reshaping the gut microbiota toward a beneficial and balanced profile.

### 3.8. Short-Chain Fatty Acid Profiling of F. plautii PMC93 in HGMS

**Figure 8:**
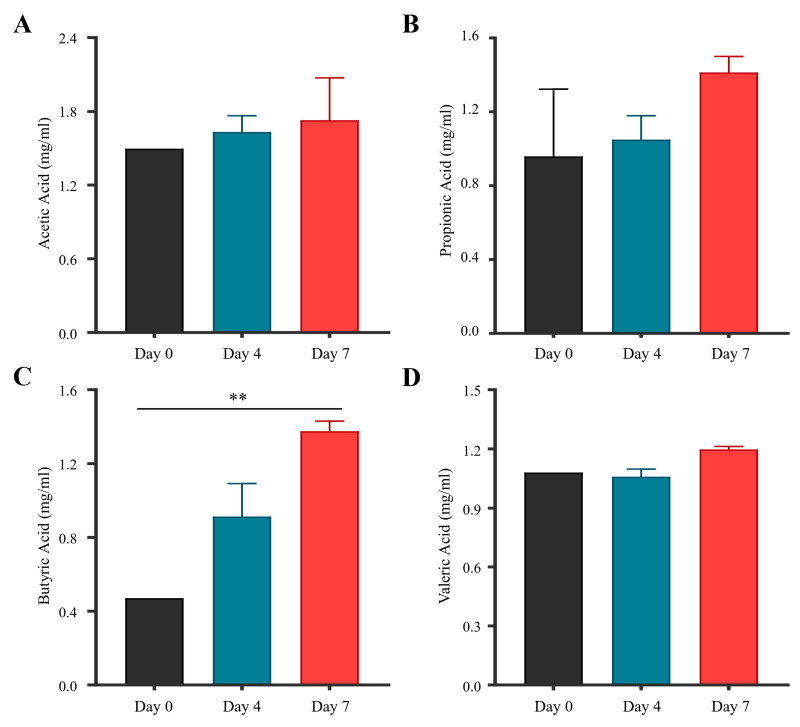
Short-chain fatty acid (SCFA) quantification after PMC93 supplementation. SCFA concentrations were measured at days 0, 4, and 7 in HGMS following PMC93 treatment. Acetic acid (**A**) concentrations exhibited a gradual but non-significant increase across time points. Propionic acid (**B**) also showed a modest rise by day 7, not reaching statistical significance. Butyric acid levels (**C**) displayed a significant increase by day 7 compared to day 0 (**, *p* < 0.01), demonstrating a marked change over time. Valeric acid levels (**D**) had a slight upward trend over the duration, with no significant differences observed. For each experimental group, three biological samples (N = 3) were analyzed. Values are reported as means with standard deviations. Statistical significance was analyzed using one-way ANOVA (GraphPad Prism 9.1.1).

SCFA analysis of HGMS samples demonstrated time-dependent alterations following continuous PMC93 treatment (Figure 8 and Table S4). Acetic acid levels progressively increased over the experimental timeline, with levels rising from day 0 to day 4 and further at day 7 (Figure 8A). Despite the observed upward trajectory, the differences among these time points did not achieve statistical significance. Propionic acid concentrations displayed a comparable pattern, exhibiting stepwise increases from day 0 to day 7 (Figure 8B). While levels at day 7 were greater than baseline, this change was not statistically significant. In contrast, butyric acid presented a significant elevation over the course of the study (Figure 8C). A substantial increase was evident from day 0 to day 7 (*p* < 0.01), with the concentration nearly doubling by the final time point. This outcome underscores a pronounced stimulatory influence of PMC93 on butyrate-producing bacteria or metabolic pathways. Valeric acid concentrations were generally stable; nevertheless, a minor increasing trend was noted at day 7 (Figure 8D). Despite this gradual elevation, statistical analysis indicated no significant differences across the time points.

### 3.9. F. plautii PMC93 Mediated In Vivo Regulation of Inflammatory Gene Expression

**Figure 9:**
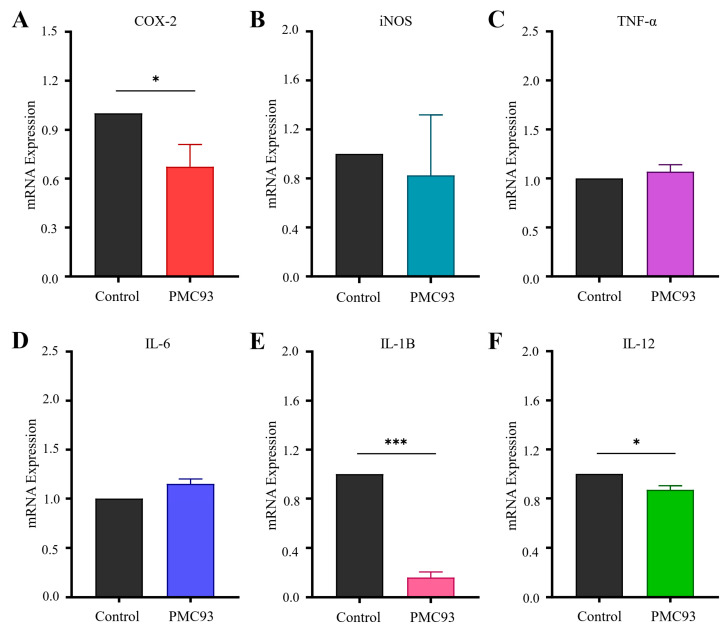
Regulation of inflammatory gene expression by PMC93. After two weeks of PMC93 administration, gut tissues from mice (each group N = 5) were collected for quantitative real-time PCR analysis of mRNA expression. (**A**) COX-2 expression was markedly reduced in the PMC93 group compared to controls, and (**B**) iNOS expression displayed a mild decrease following PMC93 treatment, although this was not statistically significant. Conversely, IL-6 and TNF-α levels (**C**,**D**) showed upward trends without reaching significance, while PMC93 administration caused a significant reduction in IL-1β and IL-12 expression (**E**,**F**). All analyses were performed in triplicate and repeated independently three times. Results are presented as means with standard deviations. Statistical significance (* p < 0.05, *** p < 0.001) was evaluated with the unpaired *t*-test (GraphPad Prism 9.1.1).

Quantitative real-time PCR analysis revealed that PMC93 administration robustly modulated the expression of inflammation-associated genes in the gut (Figure 9). Specifically, expression of COX-2 was significantly reduced in the PMC93-treated group when compared to controls (* *p* < 0.05) (Figure 9A). Although iNOS, TNF-α, and IL-6 mRNA expression showed slight elevations following PMC93 exposure, these variations were not statistically significant (Figure 9B–D). Conversely, a substantial anti-inflammatory response was observed, as PMC93 significantly repressed expression of IL-1β (*** *p* < 0.001) and IL-12 (* *p* < 0.05) relative to controls (Figure 9E,F).

### 3.10. Acute Oral Toxicity Profile of F. plautii PMC93

**Figure 10:**
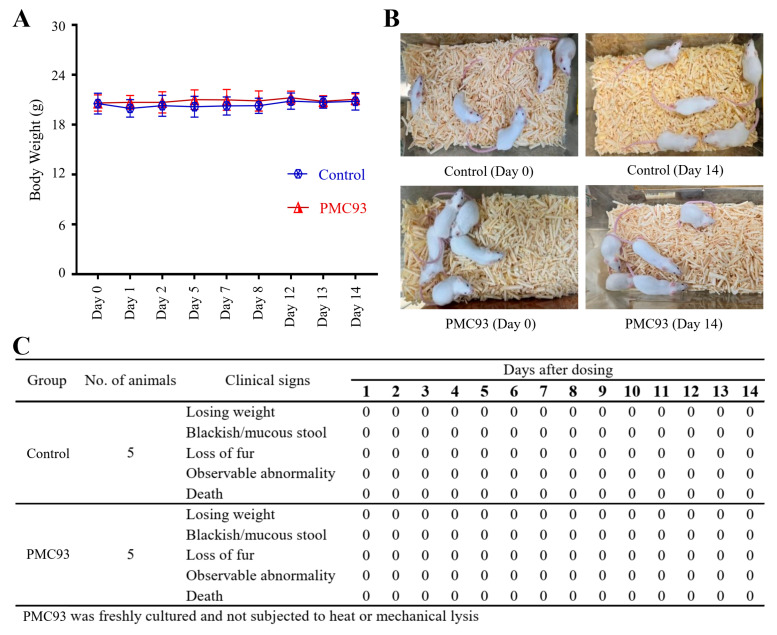
Acute oral toxicity assessment for PMC93. Freshly cultured PMC93 was administered orally (3 days/week) for a duration of two weeks in mice (each group N = 5). Body weight (**A**) was monitored over 14 days. No significant differences in body weight were detected between the PMC93-treated and control groups. Representative images of mice (**B**) from both control and PMC93 groups indicate no observable signs of distress or abnormality. Compilation of clinical observations during the 14-day treatment course (**C**) revealed that no clinical indications of toxicity appeared in either group throughout the entire study.

To evaluate the safety profile of PMC93, a repeated oral toxicity study was carried out in mice for 14 days (Figure 10). Body weight measurements were consistently taken, and animals were closely monitored for clinical symptoms. There were no statistically significant differences in body weight between the PMC93 and control group throughout the study period (Figure 10A), suggesting that PMC93 had no adverse effect on weight gain or growth trajectories. Observational assessment of the animals at both the start and end of the experiment (Figure 10B) did not reveal any behavioral or physical abnormalities. Additionally, Figure 10C shows that no subjects in either cohort manifested any signs of toxicity, such as weight loss, blackish or mucous stool, fur loss, external abnormalities, or lethality during the observation period. All mice administered with PMC93 appeared healthy and indistinguishable from those in the control group, which received only distilled water.

## 4. Discussion

Probiotics have long been recognized as important modulators of gut health, with widely used strains such as *Lactobacillus*, *Bifidobacterium*, and *Saccharomyces* demonstrating diverse health-promoting effects [48]. Their benefits include enhancement of epithelial barrier function, production of SCFAs, immunomodulation, and competitive exclusion of pathogens, thereby improving gut health and supporting host defense [48,49]. However, conventional probiotics—derived mainly from fermented foods or easily isolated from stool—have already been extensively studied and commercialized, resulting in a saturated research and application landscape that limits the potential for further breakthroughs [50]. In addition, their colonization is transient, efficacy inconsistent, and viability often limited by oxygen sensitivity [50,51,52].

In this context, attention has shifted toward NGPs, defined as commensal gut microbes that are difficult to culture, relatively recently identified, and not yet extensively studied, but with specific health-promoting properties that could enable future commercialization [53]. Pioneering NGPs such as *Akkermansia muciniphila* and *Faecalibacterium prausnitzii* have demonstrated promising roles in maintaining intestinal barrier integrity, regulating host metabolism, and suppressing inflammatory pathways [8,54]. These bacteria are considered more representative of the core gut microbiota than traditional probiotics and are potentially more effective in restoring dysbiosis-associated conditions [9,11]. Nevertheless, the transition of NGPs into clinical applications remains challenging, with most studies still restricted to preclinical or small-scale trials [55]. Cultivation and large-scale production of obligate anaerobes remain technically demanding, and their oxygen sensitivity complicates formulation and delivery [56]. Safety evaluation is also critical, as these organisms are relatively less studied in humans compared to conventional probiotics [57]. Therefore, beyond NGPs, there is growing recognition of the need to pursue “next-generation microbiome strains,” a broader concept encompassing highly fastidious, underexplored taxa with distinctive ecological and metabolic traits. Their systematic discovery and development could yield novel metabolic and immunological functions with transformative potential for microbiome-based therapeutics and the commercialization of probiotics [58].

Recent advances in microbiome research have begun to expand the repertoire of candidate taxa. Underexplored species such as *Christensenella minuta*, *Roseburia intestinalis*, and *Blautia* spp. have demonstrated promising roles in metabolic regulation and immune modulation [59,60]. In parallel, synthetic biology and microbial engineering have enabled new approaches to enhance microbial functionality and stability [61]. However, most candidates remain in the discovery or early development stage with limited clinical validation, reinforcing the importance of targeting novel, hard-to-culture next-generation microbiome strains as the future direction for the field [62].

In pursuit of such a targeted NGP, a rigorous culturomics-based strategy was undertaken, enabling the isolation of *F. plautii*, a unique and previously uncharacterized commensal bacterium with exceptional probiotic potential. The technical challenges of isolating this obligate anaerobe under strictly controlled anaerobic conditions highlight not only the rarity of this achievement but also its significance, as such taxa are often considered “uncultivable” by conventional methods [63]. Importantly, whole-genome sequencing confirmed that *F. plautii* PMC93 is a novel strain, revealing distinct genetic traits related to bile acid transformation, immune regulation, and flavonoid metabolism. Compared to the existing literature, where only a limited number of *Flavonifractor* strains have ever been described [64], our discovery marks a significant advance by establishing a previously uncharacterized member of the human gut microbiota as a strong NGP candidate. This finding carries immense academic importance, as it not only expands the spectrum of cultivable NGPs but also provides unique insights into microbial functions that have been largely overlooked in probiotic research.

To assess the intestinal effects of PMC93 under conditions closely resembling the human gut environment, we employed an artificial digestion model. This approach is critical because conventional animal models, while informative, often fail to fully replicate the complex microbial composition, metabolic interactions, and physiological parameters of the human gastrointestinal tract [65,66]. The artificial digestion model enables the controlled and reproducible simulation of the human intestinal environment, allowing for the precise monitoring of microbial community shifts, metabolic outputs, and strain-specific activities [66]. By using this human-like in vitro system, we were able to evaluate the functional impact of PMC93 in a setting that more accurately reflects its potential performance in real-world human applications [66].

One of the most important outcomes of this study is that PMC93 appeared microbiome-safe, meaning that it does not disrupt the overall microbial diversity or induce dysbiosis, even under daily administration in a simulated human gut environment. This safety aspect is critical for NGP development, as novel strains must maintain ecological stability within the gut while delivering measurable benefits [57]. The administration of PMC93 resulted in non-significant changes in gut microbial richness and the overall community structure. Indices reflecting richness, such as Chao1 and Fisher indexes, showed a slight increase in the PMC93 group relative to the control group, indicating that supplementation may have facilitated either the colonization or persistence of a greater diversity of microbial taxa [67]. Conversely, diversity indices such as Shannon and Simpson did not display comparative changes, highlighting that the relative balance between dominant species remained largely unaltered [68]. These findings imply that PMC93 was able to expand the range of taxa present without disrupting the evenness of the microbial community [68]. Beta diversity analysis provided further evidence that PMC93 induced marginal alterations in gut microbiota composition, as demonstrated by pronounced group separation in both Jensen–Shannon and Bray–Curtis metrics. Collectively, these findings demonstrate that while PMC93 may promote minor shifts in taxonomic representation, it does so without inducing dysbiosis or disrupting microbial homeostasis [69,70].

A key differentiator of PMC93 is its effect on the F/P Index. This ratio, proposed here as the NGP F/P Index, serves as an integrated marker of gut ecosystem balance and inflammatory status [71]. Elevation of this index is generally associated with improved intestinal resilience, lower levels of pro-inflammatory taxa, and enhanced metabolic capacity [72,73]. At several taxonomic tiers, the PMC93 group showed elevated levels of beneficial *Firmicutes*, particularly from the class *Clostridia*, the order *Clostridiales*, and families including *Lachnospiraceae*, *Veillonellaceae*, and *Prevotellaceae*. These microbial groups are established contributors to the synthesis of SCFAs, most notably butyrate, which reinforces intestinal barrier function, mediates anti-inflammatory pathways, and underpins host metabolic fitness [74,75,76]. In contrast, the control group harbored greater numbers of *Proteobacteria*, particularly *Gammaproteobacteria* and *Enterobacteriaceae*. Higher levels of *Proteobacteria* are commonly associated with gut dysbiosis and inflammatory conditions, as this phylum contains numerous opportunistic pathogens and endotoxin-producing bacteria [77,78,79]. The dominance of *Enterobacteriaceae* in the control group is especially significant, given that it includes such species as *Escherichia* and *Klebsiella*, which are linked to inflammation and metabolic endotoxemia [80]. Notably, the PMC93 group exhibited a marked increase in genera such as *Mitsuokella*, *Megasphaera*, *Moryella*, and *Prevotella*, most of which are recognized as SCFA producers or are known to participate in fiber fermentation [81,82]. The pronounced increases in *Megasphaera* and *Prevotella* lend further support to the hypothesis that the NGP intervention fosters a microbiota conducive to improved metabolic function [69]. At the same time, genera such as *Pseudomonas*, *Stenotrophomonas*, *Dialister*, and *Veillonella*, which were more abundant in the control group, have demonstrated variable associations with both pro-inflammatory processes and protection depending on the host environment [83,84,85].

A significant methodological advancement of this research is the establishment of the ex vivo MT-HGMS, a system designed to overcome the limitations of conventional gut simulators. Traditional HGMS models typically allow the testing of only two or three conditions at a time [32], which restricts comparative analysis and often introduces variability across experiments. In contrast, the ex vivo MT-HGMS enables the simultaneous evaluation of multiple treatments—including probiotics, drugs, and their combinations—within a single, controlled experimental setup. This innovation not only enhances experimental throughput and efficiency but also ensures that different interventions are directly compared under identical conditions, minimizing confounding factors. Using ex vivo MT-HGMS when benchmarked against conventional probiotic strains, PMC93 consistently demonstrated a superior ability to restructure the gut microbiota toward a composition associated with improved health outcomes, and unlike conventional probiotics, which produced modest or negligible effects on the F/P ratio, PMC93 treatment markedly increased this index, the highest among all groups. This increase reflected not only a substantial enrichment of beneficial *Firmicutes* (e.g., *Oscillospira*, *Faecalibacterium*) but also a pronounced suppression of potential pathobionts (notably *Pseudomonas* and *Stenotrophomonas*), all without compromising overall microbial diversity. In addition, the robustness of PMC93′s modulatory effect was further reinforced under conditions simulating combined exposure to common pharmaceuticals and functional ingredients. Across all tested co-treatment scenarios, including Vitamin D, Albendazole, Famotidine, and Finasteride, PMC93 maintained its ability to elevate the F/P Index relative to equivalent probiotic–drug combinations. Importantly, these findings align with prior literature reporting that several pharmaceuticals can depress the F/P ratio or otherwise skew gut communities toward *Proteobacteria*-dominant [86,87], pro-inflammatory states [88]; in that context, PMC93′s capacity to counteract such drug-induced shifts underscores its translational relevance. These microbiota shifts were accompanied by selective enrichment of SCFA-associated taxa, particularly *Oscillospira*, and sustained suppression of *Proteobacteria* genera linked to inflammation [89,90]. The persistence of these benefits under pharmacological co-exposure highlights PMC93′s resilience and functional stability over conventional probiotic strains in both baseline and therapeutically challenged conditions. As the study focused on probiotic comparisons, a supplement-only condition was not included, though its independent effects will be examined in future work.

This improvement was linked to functional benefits, including a measurable increase in SCFA metabolites, which are well known for their anti-inflammatory properties and ability to support gut barrier integrity. This enrichment was driven by an increase in SCFA-producing taxa, many of which belong to the *Firmicutes* phylum [91]. PMC93 treatment resulted in a significant increase in butyric acid concentrations, indicating enhanced functional activity of butyrate-producing microbial populations. Butyrate, a central SCFA, is recognized for its role in promoting gut barrier integrity and mediating anti-inflammatory responses, which implies potential health benefits from PMC93 [92,93]. Although elevations in acetic, propionic, and valeric acids were observed, these increases did not reach statistical significance. The specific augmentation of SCFA production underscores the targeted postbiotic action of PMC93 on the gut microbiome and may suggest its utility in supporting gut health [94,95]. Beyond compositional and metabolic shifts, the anti-inflammatory potential of PMC93 was supported by gene expression data indicating downregulation of inflammation-associated pathways [96]. PMC93 administration significantly modulated immune profiles associated with gut health, although the physiological relevance of these shifts requires further validation. Notably, there was substantial downregulation of pro-inflammatory cytokines COX-2, IL-1β, and IL-12, underscoring the anti-inflammatory properties of PMC93 [97]. Although TNF-α and IL-6 exhibited minor, non-significant increases, these patterns may indicate immunological homeostasis rather than excessive immune activation [98]. Its ability to consistently elevate the NGP F/P Index while enhancing SCFA production and reducing pro-inflammatory taxa sets a new benchmark for what can be expected from next-generation probiotics.

Importantly, the safety profile of PMC93 was established via a repeated oral toxicity assessment in mice, wherein neither body weight, clinical appearance, nor behavioral parameters differed significantly from those of the control group. Moreover, given its unique genomic features, targeted microbiome-modulatory capacity, resilience in diverse exposure environments, and demonstrated safety [99], PMC93 stands out as a strong candidate for development as a health functional ingredient and as a pharmaceutical material. Our current mechanistic explanation focuses on the observed functional outcomes, acknowledging that genome-level validation will be explored in subsequent studies.

To summarize, this study provides preliminary evidence that *F. plautii* PMC93 meets several key characteristics for an NGP microbiome, including safety, targeted compositional modulation, functional metabolic enhancement, and immune regulatory potential, positioning it as a promising intervention for microbiome-informed health and disease management. This aspect will be addressed in future studies to strengthen the platform. However, the findings are based on an in vitro model, which limits the direct applicability to clinical settings. Confirmation of efficacy and safety in humans requires additional in vivo research, including clinical trials.

## 5. Conclusions

**Figure 11:**
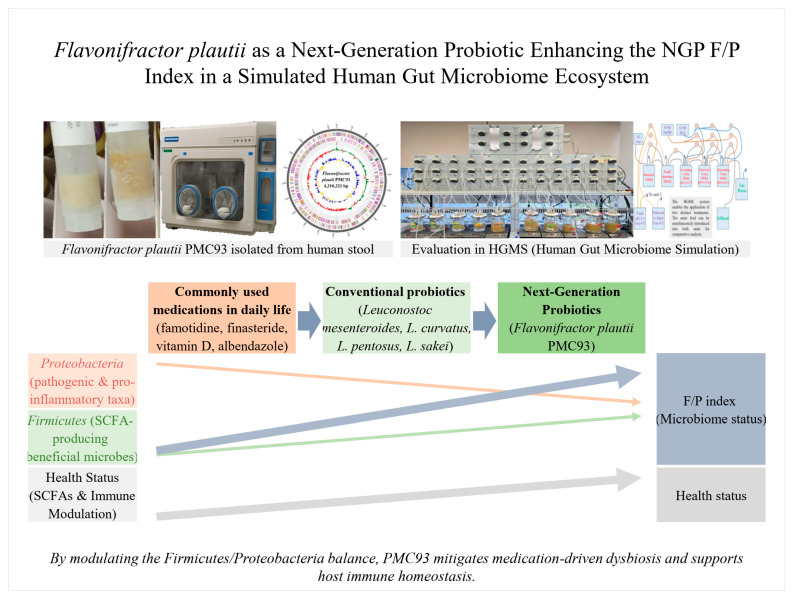
**Graphical summary.** *Flavonifractor plautii* PMC93 supplementation reshapes the gut microbiota by enhancing the NGP F/P index, a pivotal marker of microbial and intestinal health. PMC93, an obligate anaerobe isolated from human stool, was introduced into a human gastrointestinal simulator to evaluate its next-generation probiotic potential. Mechanistic evaluation revealed increased SCFA (butyrate) production, improved intestinal health function, and balanced immune modulation. Metagenomics analysis revealed its ability to promote beneficial *Firmicutes* while reducing harmful *Proteobacteria*. Unlike conventional probiotics and drug-based interventions, which typically decrease the F/P index, PMC93 induced a marked and sustained elevation, surpassing control, probiotic, and drug treatment groups. These findings position PMC93 as a promising NGP, offering a novel therapeutic avenue for restoring gut microbial balance and promoting intestinal health.

This study presents robust evidence that *F. plautii* PMC93 is a safe and well-characterized next-generation probiotic candidate, supported by comprehensive microbiome-safety profiling and taxonomic validation. PMC93 exhibited strong efficacy by modulating gut microbial composition, enhancing short-chain fatty acid production—especially butyrate, and reducing inflammation-associated *Proteobacteria*, collectively improving the NGP F/P Index. Its functional benefits remained consistent and stable across multiple conditions, including co-exposure to pharmaceutical agents, highlighting its robustness and resilience within the gut ecosystem. Together, these findings position PMC93 as a promising microbial candidate for further preclinical development and potential clinical translation. The comprehensive workflow and findings were summarized in Figure 11 to provide a foundation for future microbiome-based research and practical applications.

## Figures and Tables

**Table 1:** 16s rRNA genome sequencing and BLAST analysis results in relation to corresponding NCBI records. NCBI, National Center for Biotechnology Information.

NCBI Reference	Organism	Length	Score	Identities	Gaps
NR_029356.1	*Flavonifractor plautii* strain 265	1550	2673 bits (1447)	1453/1456 (99%)	0/1456 (0%)
NR_043142.1	*Flavonifractor plautii* strain Prevot S1	1465	2634 bits (1426)	1440/1447 (99%)	0/1447 (0%)
NR_147370.1	*Pseudoflavonifractor phocaeensis* strain P3064	1495	2475 bits (1340)	1417/1456 (97%)	0/1456 (0%)
NR_180619.1	*Pseudoflavonifractor gallinarum* strain Cla-CZ	1433	2457 bits (1330)	1389/1418 (98%)	1/1418 (0%)
NR_025670.1	*Pseudoflavonifractor capillosus* ATCC 29799	1483	2457 bits (1330)	1413/1454 (97%)	1/1454 (0%)
NR_179410.1	*Clostridium phoceensis* strain GD3	1495	2440 bits (1321)	1411/1456 (97%)	0/1456 (0%)
NR_173697.1	*Lawsonibacter asaccharolyticus* strain 3BBH22	1481	2399 bits (1299)	1405/1457 (96%)	3/1457 (0%)
NR_179423.1	*Intestinimonas timonensis* strain GD4	1495	2346 bits (1270)	1395/1457 (96%)	2/1457 (0%)
NR_179418.1	*Intestinimonas massiliensis* strain GD2	1519	2266 bits (1227)	1382/1458 (95%)	5/1458 (0%)

**Table 2:** Summary of average taxonomic composition with statistically significant differences. Taxonomic relative abundances (%) are reported only for taxa comprising more than 1% of the community and demonstrating statistical significance (* *p* < 0.05; *** *p* < 0.001) with respect to the Wilcoxon rank-sum test.

Phylum	Control	PMC93	Class	Control	PMC93	Order	Control	PMC93	Family	Control	PMC93	Genus	Control	PMC93
*Bacteroidetes*	33.4	32.8	*Bacteroidia*	33.4	32.8	*Bacteroidales*	33.4	32.8	*Bacteroidaceae* ***	33.4	32.8	*Bacteroides* ***	25.7	18.4
									*Porphyromonadaceae* ***	1.9	1	*Parabacteroides* ***	1.9	1
									*Prevotellaceae* ***	1.9	9.9	*Prevotella* ***	1.9	9.9
*Firmicutes* ***	24.7	43.3	*Clostridia* ***	23.7	43.1	*Clostridiales* ***	23.7	43.1	*Lachnospiraceae* ***	4.2	9.3	*Clostridium* ***	1.6	3.2
												*Moryella* ***	0.7	4.4
									*Veillonellaceae* ***	16.2	31.6	*Dialister* ***	1.2	0.4
												*Megasphaera* ***	0	2.9
												*Veillonella* ***	4	0.7
												*Mitsuokella* ***	10.8	26.8
*Proteobacteria* ***	41	23.5	*Desulfovibrionia* *	0.9	1.3	*Desulfovibrionales* ***	0.9	1.3	*Desulfovibrionaceae* ***	0.9	1.3	*Bilophila* *	0.9	1.3
			*Alphaproteobacteria* ***	0.5	2.1	*Caulobacterales* ***	0.5	2.1	*Caulobacteraceae* ***	0.5	2.1	*Nitrobacteria* ***	0.5	2.1
			*Gammaproteobacteria* ***	39.4	20	*Enterobacteriales* ***	16.6	1.5	*Enterobacteriaceae* ***	16.6	1.5			

## Data Availability

The data supporting the conclusions of this article will be made available by the authors on request.

## Supplementary Materials

The following supporting information can be downloaded at: https://www.mdpi.com/article/10.3390/pharmaceutics17121603/s1, Table S1: 16S rRNA genome sequences of conventional probiotics and corresponding BLAST analysis results; Table S2: Taxonomic profile of the samples; Table S3: Primer sets for pro-inflammatory and housekeeping gene analysis [1,100,101,102]; Table S4. Short-chain fatty acid (SCFA) profiling on day 7.
