# Supplementary material for: Flavonifractor plautii as a Next-Generation Probiotic Enhancing the NGP F/P Index in a Simulated Human Gut Microbiome Ecosystem
Source: Pharmaceutics. 2025 Dec 12;17(12):1603. doi: 10.3390/pharmaceutics17121603 (PMC12736906; doi:10.3390/pharmaceutics17121603)
Supplement: Supplementary file 1 [file pharmaceutics-17-01603-s001.zip › pharmaceutics-3994224-supplementary.pdf]

Supplementary Information

Table S1. 16S rRNA genome sequences of conventional probiotics and corresponding BLAST analysis results.

Organism	NCBI Reference	Strains	Length	Identities	Gaps
Latilactobacillus sakei	NR_113821.1	Latilactobacillus sakei strain NBRC 15893	1499	1457/1457 (100%)	0/1457 (0%)
	NR_042443.1	Latilactobacillus sakei strain DSM 20017	1561	1459/1460 (99%)	0/1460 (0%)
	NR_042437.1	Latilactobacillus curvatus strain DSM 20019	1559	1449/1461 (99%)	4/1461 (0%)
	NR_042438.1	Latilactobacillus graminis strain G90	1548	1447/1460 (99%)	2/1460 (0%)
Lactiplantibacillus pentosus	NR_029133.1	Lactiplantibacillus pentosus strain 124-2	1519	1485/1485 (100%)	0/1485 (0%)
	NR_113338.1	Lactiplantibacillus plantarum strain NBRC	1492	1484/1485 (99%)	0/1485 (0%)
	NR_115605.1	Lactiplantibacillus plantarum strain JCM 1149	1519	1483/1485 (99%)	0/1485 (0%)
	NR_025447.1	Lactiplantibacillus paraplantarum strain DSM	1502	1484/1487 (99%)	0/1487 (0%)
Leuconostoc mesenteroides	NR_074957.1	Leuconostoc mesenteroides strain ATCC 8293	1549	1438/1439 (99%)	0/1439 (0%)
	NR_157602.1	Leuconostoc mesenteroides strain DRC1506	1551	1437/1439 (99%)	0/1439 (0%)
	NR_113912.1	Leuconostoc mesenteroides strain NBRC 100496	1476	1433/1434 (99%)	0/1434 (0%)
	NR_113254.1	Leuconostoc mesenteroides strain JCM 9700	1473	1433/1434 (99%)	0/1434 (0%)
Latilactobacillus curvatus	NR_042437.1	Latilactobacillus curvatus strain DSM 20019	1559	1463/1469 (99%)	2/1469 (0%)
	NR_113334.1	Latilactobacillus curvatus strain NBRC 15884	1491	1456/1460 (99%)	3/1460 (0%)
	NR_042438.1	Latilactobacillus graminis strain G90	1548	1461/1469 (99%)	3/1469 (0%)
	NR_042443.1	Latilactobacillus sakei DSM 20017	1561	1456/1472 (99%)	5/1472 (0%)

NCBI, National Center for Biotechnology Information.

Table S2. Taxonomic profile of the samples.

Taxonomic rank	Taxon designation	Control							PMC93						
		Ave	SD	Max	Min	Q1	Q2	Q3	Ave	SD	Max	Min	Q1	Q2	Q3
Phylum	Actinobacteria	0.7	0.3	1.2	0.4	0.5	0.6	0.8	0.1	0.1	0.2	0.0	0.0	0.1	0.1
	Bacteroidetes	33.4	1.0	35.0	31.8	32.6	33.6	33.9	32.8	1.7	34.9	30.9	31.3	32.6	34.6
	Firmicutes	24.7	4.5	29.6	19.9	20.7	24.5	29.0	43.3	3.0	47.4	40.0	40.8	43.2	45.5
	Lentisphaerae	0.2	0.1	0.3	0.1	0.1	0.1	0.2	0.2	0.1	0.5	0.1	0.2	0.2	0.3
	Proteobacteria	41.0	3.7	45.3	36.2	37.9	41.2	44.6	23.5	1.6	25.6	20.9	22.6	23.8	24.8
Class	Actinobacteria	0.7	0.3	1.2	0.4	0.5	0.6	0.8	0.1	0.1	0.2	0.0	0.0	0.1	0.1
	Alphaproteobacteria	0.5	0.1	0.6	0.4	0.4	0.5	0.5	2.1	0.5	3.1	1.4	1.8	2.0	2.3
	Bacilli	0.9	0.5	1.6	0.4	0.4	0.9	1.4	0.0	0.0	0.1	0.0	0.0	0.0	0.0
	Bacteroidia	33.4	1.0	35.0	31.8	32.6	33.6	33.9	32.8	1.7	34.9	30.9	31.3	32.6	34.6
	Betaproteobacteria	0.3	0.1	0.6	0.2	0.2	0.3	0.4	0.1	0.1	0.3	0.1	0.1	0.1	0.1
	Clostridia	23.7	5.1	29.1	18.5	19.2	23.5	28.6	43.1	2.9	47.0	39.9	40.7	43.0	45.3
	Deltaproteobacteria	0.9	0.2	1.2	0.5	0.7	0.9	1.1	1.3	0.4	1.9	0.8	0.9	1.4	1.6
	Gammaproteobacteria	39.4	3.4	43.3	34.9	36.6	39.6	42.5	20.0	2.3	22.5	17.2	18.1	20.1	22.1
	Lentisphaeria	0.2	0.1	0.3	0.1	0.1	0.1	0.2	0.2	0.1	0.5	0.1	0.2	0.2	0.3
Order	Bacteroidales	33.4	1.0	35.0	31.8	32.6	33.6	33.9	32.8	1.7	34.9	30.9	31.3	32.6	34.6
	Bifidobacteriales	0.7	0.3	1.2	0.4	0.5	0.6	0.8	0.1	0.1	0.2	0.0	0.0	0.1	0.1
	Burkholderiales	0.3	0.1	0.6	0.2	0.2	0.3	0.4	0.1	0.1	0.3	0.1	0.1	0.1	0.1
	Caulobacterales	0.5	0.1	0.6	0.4	0.4	0.5	0.5	2.1	0.5	3.1	1.4	1.7	2.0	2.3
	Clostridiales	23.7	5.1	29.1	18.5	19.2	23.5	28.6	43.1	2.9	47.0	39.9	40.7	43.0	45.3
	Desulfovibrionales	0.9	0.2	1.2	0.5	0.7	0.9	1.1	1.3	0.4	1.9	0.8	0.9	1.4	1.6
	Enterobacteriales	16.6	4.1	21.8	12.1	12.7	16.7	20.0	1.5	0.3	2.1	1.1	1.2	1.4	1.8
	Pseudomonadales	19.6	0.8	21.1	18.5	19.2	19.5	19.6	16.4	4.1	20.6	11.6	12.7	16.6	20.2
	Victivallales	0.2	0.1	0.3	0.1	0.1	0.1	0.2	0.2	0.1	0.5	0.1	0.2	0.2	0.3
	Xanthomonadales	3.2	0.4	3.8	2.6	3.0	3.0	3.6	2.1	1.5	3.8	0.6	0.6	1.9	3.5
Family	Alcaligenaceae	0.2	0.1	0.5	0.1	0.1	0.2	0.3	0.1	0.1	0.2	0.0	0.1	0.1	0.1
	Bacteroidaceae	25.7	1.6	27.9	23.7	24.4	25.7	27.0	18.4	1.9	20.5	16.4	16.7	18.2	20.3
	Bifidobacteriaceae	0.7	0.3	1.2	0.4	0.5	0.6	0.8	0.1	0.1	0.2	0.0	0.0	0.1	0.1
	Caulobacteraceae	0.5	0.1	0.6	0.4	0.4	0.5	0.5	2.1	0.5	3.1	1.4	1.7	2.0	2.3
	Clostridiaceae	0.0	0.0	0.1	0.0	0.0	0.0	0.0	0.0	0.0	0.0	0.0	0.0	0.0	0.0
	Desulfovibrionaceae	0.9	0.2	1.2	0.5	0.7	0.9	1.1	1.3	0.4	1.9	0.8	0.9	1.4	1.6
	Enterobacteriaceae	16.6	4.1	21.8	12.1	12.7	16.7	20.0	1.5	0.3	2.1	1.1	1.2	1.4	1.8
	Lachnospiraceae	4.2	0.3	4.5	3.8	4.0	4.3	4.4	9.3	0.4	10.0	8.7	9.1	9.3	9.5
	Paraprevotellaceae	3.7	0.8	5.1	2.7	3.0	3.6	4.0	2.9	0.3	3.4	2.5	2.7	2.9	3.2
	Porphyromonadaceae	1.9	0.4	2.6	1.3	1.6	1.8	2.1	1.0	0.1	1.2	0.8	0.9	1.0	1.1
	Prevotellaceae	1.9	0.6	2.9	1.1	1.4	2.0	2.3	9.9	3.5	13.8	6.4	6.6	9.7	13.3
	Pseudomonadaceae	19.6	0.8	21.1	18.5	19.2	19.5	19.6	16.4	4.1	20.6	11.6	12.7	16.6	20.2
	Rikenellaceae	0.2	0.1	0.4	0.1	0.1	0.2	0.2	0.5	0.2	0.8	0.3	0.3	0.5	0.8
	Ruminococcaceae	2.6	0.9	3.8	1.6	1.7	2.6	3.3	2.1	0.7	2.9	1.3	1.4	2.0	2.7
	Veillonellaceae	16.2	6.0	22.2	10.4	10.6	16.0	21.9	31.6	1.9	34.5	29.1	30.0	31.3	33.0
	Victivallaceae	0.2	0.1	0.3	0.1	0.1	0.1	0.2	0.2	0.1	0.5	0.1	0.2	0.2	0.3
	Xanthomonadaceae	3.2	0.4	3.8	2.6	3.0	3.0	3.6	2.1	1.5	3.8	0.6	0.6	1.9	3.5
Genus	Alistipes	0.2	0.1	0.4	0.1	0.1	0.2	0.2	0.5	0.2	0.8	0.3	0.3	0.5	0.8
	Bacteroides	25.7	1.6	27.9	23.7	24.4	25.7	27.0	18.4	1.9	20.5	16.4	16.7	18.2	20.3
	Bifidobacterium	0.7	0.3	1.2	0.4	0.5	0.6	0.8	0.1	0.1	0.2	0.0	0.0	0.1	0.1
	Bilophila	0.9	0.2	1.2	0.5	0.7	0.9	1.1	1.3	0.4	1.9	0.8	0.9	1.4	1.6
	Blautia	0.0	0.0	0.1	0.0	0.0	0.0	0.0	0.0	0.0	0.1	0.0	0.0	0.0	0.1
	Clostridium	1.6	0.5	2.2	1.1	1.2	1.6	2.1	3.2	0.5	4.0	2.6	2.8	3.1	3.6
	Coprococcus	0.3	0.2	0.6	0.1	0.2	0.3	0.4	0.2	0.1	0.3	0.1	0.1	0.2	0.2
	Dialister	1.2	0.1	1.4	1.1	1.1	1.2	1.3	0.4	0.1	0.6	0.2	0.2	0.4	0.5
	Faecalibacterium	0.7	0.2	1.1	0.5	0.5	0.6	0.8	0.2	0.1	0.4	0.1	0.1	0.2	0.3
	Gemmiger	0.2	0.1	0.3	0.2	0.2	0.2	0.2	0.2	0.1	0.4	0.2	0.2	0.2	0.3
	Hespellia	0.0	0.1	0.2	0.0	0.0	0.0	0.1	0.0	0.0	0.0	0.0	0.0	0.0	0.0
	Megasphaera	0.0	0.0	0.0	0.0	0.0	0.0	0.0	2.9	1.0	4.0	1.7	2.1	2.9	3.8
	Mitsuokella	10.8	6.2	17.1	4.4	5.0	10.8	16.6	26.8	2.3	30.6	23.9	25.0	26.5	28.5
	Moryella	0.7	0.1	0.9	0.5	0.6	0.7	0.8	4.4	0.6	5.1	3.3	4.0	4.4	4.9
	Nitrobacteria	0.5	0.1	0.6	0.4	0.4	0.5	0.5	2.1	0.5	3.1	1.4	1.7	2.0	2.3
	Oribacterium	0.8	0.3	1.2	0.5	0.6	0.8	1.1	0.6	0.2	0.9	0.3	0.5	0.6	0.7
	Oscillospira	1.3	0.6	2.1	0.7	0.8	1.3	1.7	1.3	0.4	1.7	0.7	0.9	1.3	1.7
	Parabacteroides	1.9	0.4	2.6	1.3	1.6	1.8	2.1	1.0	0.1	1.2	0.8	0.9	1.0	1.1
	Paraprevotella	3.7	0.8	5.1	2.7	3.0	3.6	4.0	2.9	0.3	3.4	2.5	2.7	2.9	3.2
	Phascolarctobacterium	0.2	0.0	0.2	0.1	0.1	0.1	0.2	0.8	0.2	1.1	0.5	0.6	0.8	1.0
	Prevotella	1.9	0.6	2.9	1.1	1.4	2.0	2.3	9.9	3.5	13.8	6.4	6.6	9.7	13.3
	Pseudomonas	18.7	0.9	19.6	17.3	17.8	19.1	19.4	16.3	4.1	20.6	11.4	12.5	16.4	20.1
	Roseburia	0.1	0.1	0.2	0.0	0.0	0.1	0.2	0.4	0.2	0.7	0.1	0.2	0.3	0.5
	Shuttleworthia	0.0	0.0	0.1	0.0	0.0	0.0	0.0	0.0	0.0	0.0	0.0	0.0	0.0	0.0
	Stenotrophomonas	3.2	0.4	3.8	2.6	3.0	3.0	3.6	2.1	1.5	3.8	0.6	0.6	1.9	3.5
	Sutterella	0.2	0.1	0.5	0.1	0.1	0.2	0.3	0.1	0.1	0.2	0.0	0.1	0.1	0.1
	Veillonella	4.0	0.4	4.6	3.5	3.8	4.0	4.2	0.7	0.4	1.3	0.3	0.4	0.6	1.1
	Victivallis	0.2	0.1	0.3	0.1	0.1	0.1	0.2	0.2	0.1	0.5	0.1	0.2	0.2	0.3

Table S3. Primer sets for pro-inflammatory and housekeeping gene analysis.

Gene Name	Primer	Primer Sequence
TNF-α	Forward	CGA GTG ACA AGC CCG TAG CC
	Reverse	GGA TGA ACA CGC CAG TCG CC
iNOS	Forward	CAG TGC CAC GTC ACC AACG
	Reverse	GCG CAC TGG GTC ATG ACAC
Cox-2	Forward	GGA CAT TTA GCG TCC CTG CA
	Reverse	GAG TTC CTG GAC GTG CTC CT
IL-1β	Forward	CCA GGA TGA GGA CCC AAG CA
	Reverse	TCC CGA CCA TTG CTG TTT CC
IL-6	Forward	CTT CCA GCC AGT TGC CTT CT
	Reverse	GAC AGC ATT GGA AGT TGG GG
IL-12	Forward	TTG CCC TCC TAA ACC ACC TCA
	Reverse	CTT GCT CTT CTG CTA ACA CAT
β-actin	Forward	ATG GAT GAC GAT ATC GCT
	Reverse	TGG ACT GTC TGA TGG AGTA

Sources of the above-mentioned gene primers:

TNF-α, IL-1β, IL-6,* and *β-actin - Salazar-Montes, A., V. Delgado-Rizo, and J. Armendáriz-Borunda, Differential gene expression of pro-inflammatory and anti-inflammatory cytokines in acute and chronic liver injury. Hepatology Research, 2000. 16(3): p. 181-194.

iNOS - Barman, I., et al., Isolation of New Strains of Lactic Acid Bacteria from the Vaginal Microbiome of Postmenopausal Women and their Probiotic Characteristics. Current Microbiology, 2025. 82(2): p. 76.

Cox-2 - Newton, R., et al., Evidence for involvement of NF-κB in the transcriptional control of COX-2 gene expression by IL-1β. Biochemical and biophysical research communications, 1997. 237(1): p. 28-32.

IL-12 - Coutelier, J.-P., J. Van Broeck, and S.F. Wolf, Interleukin-12 gene expression after viral infection in the mouse. Journal of Virology, 1995. 69(3): p. 1955-1958.

Table S4. Short-chain fatty acid (SCFA) profiling on day 7.

**Unit: mg/mL**	**Control**	**PMC93**
Acetic acid	1.462 ± 0.083	1.729 ± 0.344
Propionic acid	0.976 ± 0.042	1.414 ± 0.087
Butyric acid	0.721 ± 0.314	1.377 ± 0.055
Valeric acid	1.150 ± 0.056	1.198 ± 0.016

All values are presented as mean ± standard deviation (SD).
